# Mass spectrometric snapshots for electrochemical reactions†
†Electronic supplementary information (ESI) available. See DOI: 10.1039/c6sc01978a



**DOI:** 10.1039/c6sc01978a

**Published:** 2016-07-06

**Authors:** Ran Qiu, Xin Zhang, Hai Luo, Yuanhua Shao

**Affiliations:** a Beijing National Laboratory for Molecular Sciences , College of Chemistry and Molecular Engineering , Peking University , Beijing 100871 , China . Email: hluo@pku.edu.cn ; Email: yhshao@pku.edu.cn

## Abstract

A hybrid ultramicroelectrode containing one micro-carbon electrode and one empty micro-channel was employed to be a micro-electrochemical cell and a mass spectrometric nanospray emitter. This setup can combine MS with an electrode directly and provide *in situ* information about an electrochemical reaction. The mechanisms proposed by Bard *et al.* for a Ru(bpy)_3_^2+^ (bpy = 2,2′-bipyridine) electrochemiluminescence (ECL) system were confirmed by the MS detection of key intermediates. The short-lived diimine intermediate of electrochemical oxidation of uric acid was also detected, which affirms that the novel technique is able to catch fleeting intermediates. These experimental results demonstrate that this new method is simple, easy to implement and can be coupled with many commercial mass spectrometric instruments to provide very useful information about electrochemical reactions.

## Abstract 2

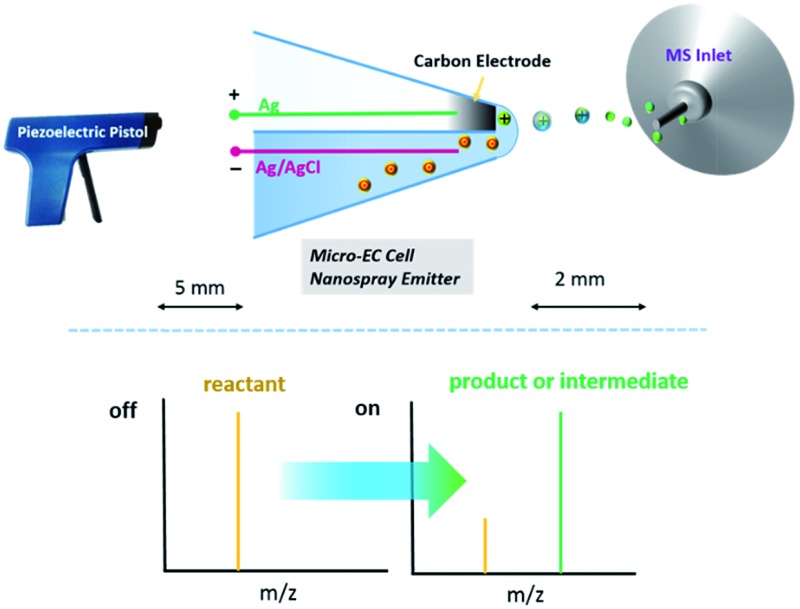
A hybrid microelectrode can connect MS and electrochemical reactions, providing direct information on products or intermediates to identify the reaction mechanisms with easy use and low cost.

## Introduction

The investigation of electrochemical reactions, especially their mechanisms, is usually carried out by typical electrochemical techniques coupled with spectroscopy,^1^ surface analysis techniques^2^ and mass spectrometry (MS).^3^ The combination of electrochemistry with MS (EC/MS) was first realized by Bruckenstein *et al.* ^4^ in 1971. Since then, with the development of novel ionization methods, EC/MS techniques have been developed quickly and applied in many areas, such as the characterization of EC reactions,^3^ drug metabolism,^5^ and biomolecules,^6–8^ as well as online derivatization of functional groups^9^ and chemical imaging.^10^

Many of the EC/MS setups were constructed *via* a flowing electrochemical cell connected to an inlet of MS; the products or intermediates generated on the electrode could not be detected by MS immediately.^11–14^ EC/MS with a faster response time to detect short-lived intermediates is challenging. Most EC coupled ambient ionization MS techniques typically have response time ranges from 0.1 to a few seconds.^15,16^ The development of DESI and easy ambient sonic-spray ionization (EASI) reduced the sampling time in the order of milliseconds.^17^ Recently, Liu *et al.* ^18^ have established a paper based electrochemical cell which can be coupled directly to a mass spectrometer, and reaction products as well as intermediates can be detected. Brown *et al.* ^19–21^ have built a water-wheel shaped electrochemical cell, and by using DESI for sampling and ionization, fleeting intermediates in electro-oxidation reactions can be detected.

Since the first introduction of dual barrel micropipettes as a probe for scanning electrochemical microscopy (SECM) by Bard *et al.*,^22^ the applications of dual micro- and nanopipettes have never faded as time has passed. They have been used to study ion transfer reactions at a liquid/liquid interface.^23,24^ With one barrel filled with pyrolytically deposited carbon, the hybrid nanopipette has been used as a probe in scanning ion conductance microscopy (SICM) to do cell surface imaging.^25^ Moreover, recently the dual barrel pipette has been applied as a nanospray emitter to do *in situ* mixing within a Taylor cone and fast reaction kinetics have been studied by MS.^26–29^

**Fig. 1:**
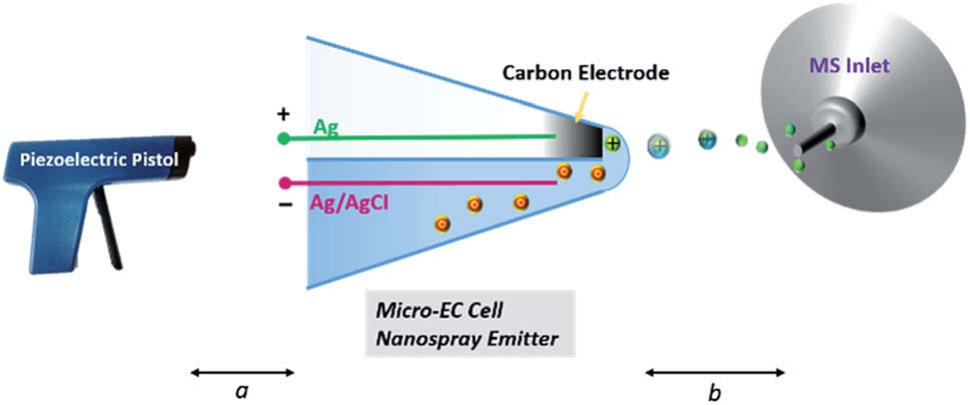
Schematic illustration of the setup. After loading the analyte solution, Ag and AgCl electrodes are inserted into the two barrels, respectively. The electrochemical cell/nanospray emitter is established. A piezoelectric pistol is used to generate primary ions. Primary ions induce spray at the tip end, and products or intermediates of a redox reaction on the carbon electrode surface can be analysed by MS. The configuration of the tip end is magnified for clarity. Distances: *a* = 5 mm, *b* = 2 mm.

Herein, a hybrid ultramicroelectrode, fabricated based on a quartz theta micropipette and the pyrolysis of butane,^25^ was employed as both the electrode for electrochemical reactions (carbon electrode) and MS nanospray emitter (empty channel filled with reactive species) as shown in Fig. 1. In this design, as the glass capillary is hydrophilic, a thin liquid layer can form at the tip of the hybrid electrode, connecting the two barrels. Consequently, a micro-electrochemical cell is established. When potential on the micro-electrochemical cell is turned on and the piezoelectric pistol^30^ starts to pump, the products and intermediates of EC reactions can be directly sampled from the carbon electrode surface and then analyzed by MS in real time. The fabrication of the hybrid ultramicroelectrode is shown in Fig. S1 of the ESI.†

The compatibility of an EC cell with electrospray ionization (ESI) is usually a challenging issue. The high voltage added in ESI might affect the low potential applied in the EC system. Li *et al.* ^30^ have recently reported a new ionization method, in which a hand-triggered piezoelectric pistol was used to generate primary ion flow, and the ions could induce spray from the capillary tip end. This new ionization strategy has negligible influence on the EC system. As shown in Fig. 1, by triggering the piezoelectric pistol, the ultra-small droplet (approximately several fLs) on the tip end of the micro-electrochemical cell can be sampled, ionized and then analyzed by MS.

## Experimental

### Chemicals and reagents

Dopamine (99%), uric acid (99%), ammonium chloride (99.5%), potassium chloride (99.5%) and methanol (99%) were obtained from Sigma Aldrich (St. Louis, MO, USA). Pure water was purchased from Wahaha Company (Hangzhou, Zhejiang, China). Silver wire (diameter 0.3 mm) was obtained from Alfa Aesar (Shanghai, China) and used as an Ag electrode. The Ag/AgCl electrode was made after electro-deposition of the silver wire in saturated KCl solution for 3 h. All chemicals were of analytical grade or higher and were used as received.

### Fabrication of the hybrid ultramicroelectrodes

Dual pipettes were fabricated from quartz theta capillaries (1.2 mm outer diameter, 0.90 mm inner diameter) using a laser-based P-2000 pipette puller (Sutter Instrument Co., Novato, CA, USA). One barrel of the dual pipettes was blocked by clay, butane was passed through the other barrel of the dual pipettes. The tip of the pipette was heated by an alcohol lamp for 30 s under a nitrogen atmosphere, to pyrolytically deposit carbon from the butane, as illustrated in Fig. S1.† Electrical contact to both electrodes was established by inserting a silver wire (carbon electrode) and Ag/AgCl (solution filled channel). The diameter of the hybrid ultramicroelectrode is about 2 μm. Two barrels evenly share the outlet of the tip end. The parameters of the P-2000 laser puller for fabrication of dual micropipettes are below:

Line 1, H:730, F:4, V:20, D:140, P:60;

Line 2, H:670, F:3, V:40, D:130, P:90.

In this way, we can obtain two almost identical dual micropipettes in one pulling operation.

### Mass spectrometry

MS experiments were carried out using a Thermo Finnigan LCQ Advantage MAX ion trap mass spectrometer (San Jose, CA, USA). The main experimental parameters were as follows: spray voltage 0 kV, capillary voltage 4.0 V, tube lens offset –50.0 V, and heated capillary temperature 250 °C. All mass spectra were acquired in positive or negative mode with 3 microscans and were recorded by instrumental software (Xcalibur version 1.4 SR1). The piezoelectric pistol was a generous gift from Prof. R. Graham Cooks and Dr Anyin Li (Purdue University). The pistol was triggered by hand and used to induce spray from the tip end of the hybrid electrode. Each trigger would cause an MS signal to last about 2 seconds. Applied voltage was controlled by a CHI 910B electrochemical workstation (CHI Instruments Inc., TA, USA).

## Results and discussion

**Fig. 2:**
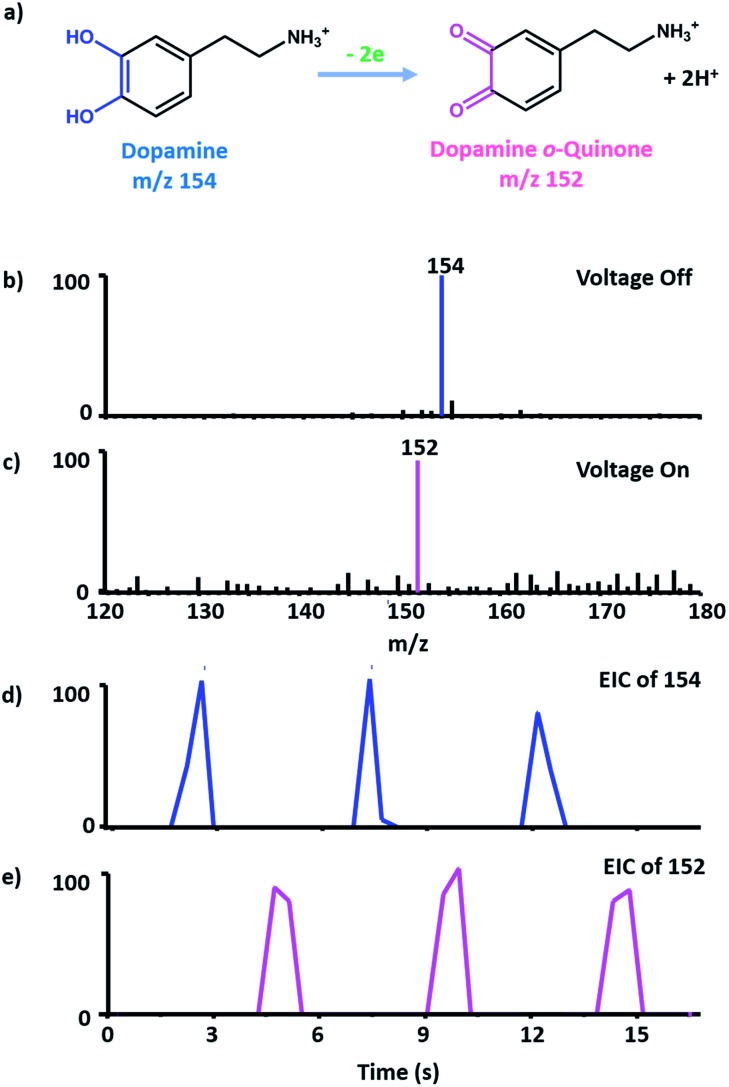
(a) Dopamine oxidation reaction. (b) Mass spectrum when no potential is applied. (c) Mass spectrum when 1.0 V is applied. (d and e) 6 triggers are continuously shot. The 1st, 3rd, and 5th triggers are shot when the potential is off. The 2nd, 4th, and 6th triggers are shot when the voltage is on. (d) The extracted ion chromatogram (EIC) of *m*/*z* 154 during the 6 shots. (e) The EIC of *m*/*z* 152 during the 6 shots.

Dopamine (DA) is an important neurotransmitter, and also a well-studied molecule in electrochemistry.^31^ Here it was chosen as an example for proof of concept, and the oxidation process of DA to dopamine *o*-quinone has been investigated by cyclic voltammetry (CV) using the micro-electrochemical cell and the EC/MS setup. The electrochemical response of 2 mM DA in the hybrid electrode is a well-defined voltammogram (see Fig. S2†), which confirms that the micro-electrochemical cell works properly. Then the EC/MS experiment was carried out, 10 ppm DA solution (10 mM NH_4_Cl as the supporting electrolyte) was injected into the lower barrel shown in Fig. 1. When no potential was applied on the EC system, the molecular ion of DA (*m*/*z* 154) was detected (Fig. 2b). When the potential of 1.0 V was controlled precisely by a potentiostat, the oxidized product dopamine *o*-quinone could be detected as *m*/*z* 152 (Fig. 2c), and the *m*/*z* 154 peak corresponding to DA was absent. This demonstrates that the EC/MS setup functions well. Interestingly, if the applied potential was on and a single pulse by the piezoelectric pistol was triggered, the MS signal of dopamine *o*-quinone appeared without that of the reactant (DA). If the potential was switched off, and then a single pulse was triggered, the MS signal of DA arose and no oxidized products could be obtained in the mass spectrum. When the potential was turned on again, only the oxidized product could be obtained through one trigger (see the extracted ion chromatogram in Fig. 2d and e). These results indicate that the thin liquid layer on the tip can be sampled completely and refreshed (also see notes in Fig. S3† for details). They also demonstrate that the electrochemical reactions on the ultramicroelectrode surface can be monitored successfully and instantaneously by MS with good reproducibility. Fig. 2 also indeed shows that the effect of the piezoelectric pistol on electrochemical reactions is rather limited.

**Fig. 3:**
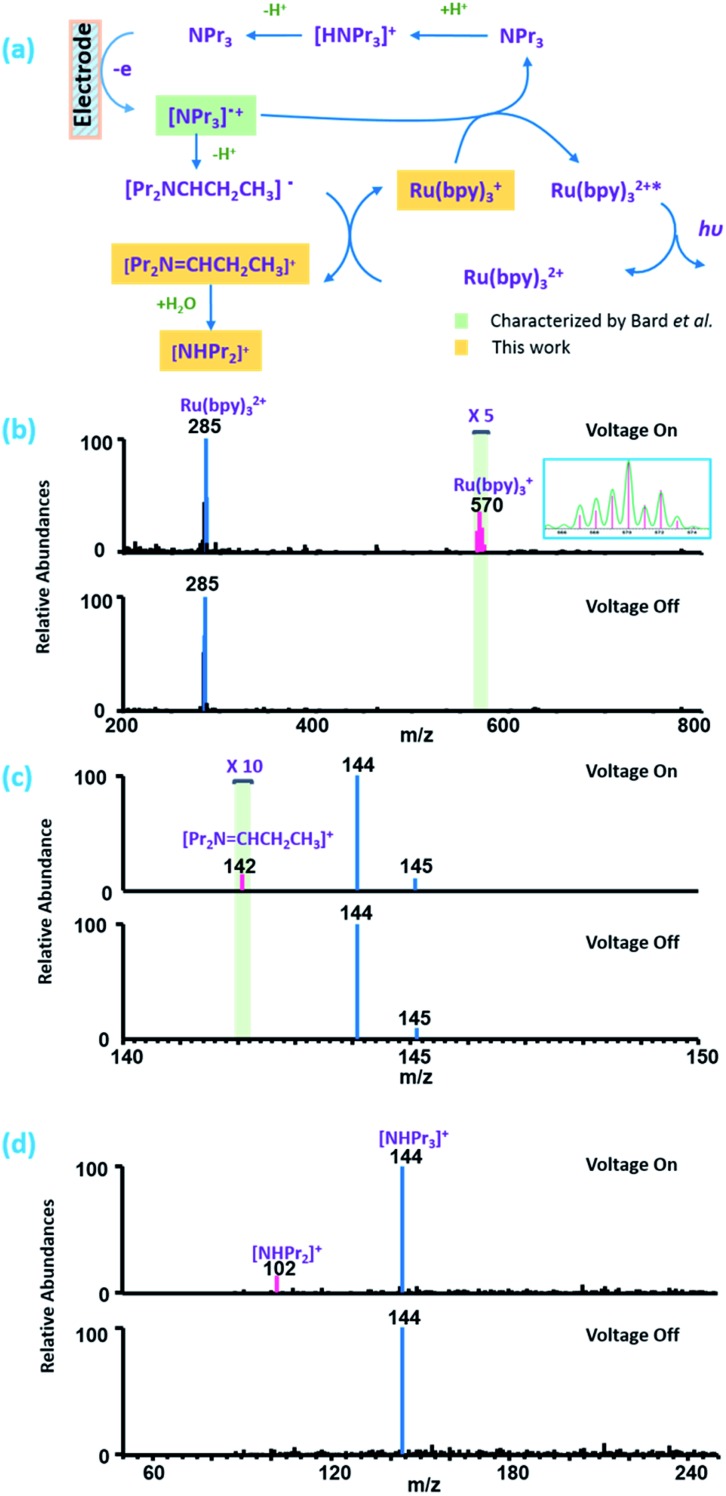
(a) Mechanistic route of ruthenium(ii) electrochemiluminescence when a voltage of 0.8 V is applied. (b) Detection of the Ru(bpy)_3_^+^ ion. The inset is the isotopic distributions of Ru(bpy)_3_^+^ (detected in pink, theoretical in green). (c) Detection of the [Pr_2_N = CHCH_2_CH_3_]^+^ intermediate. (d) Detection of the [NHPr_2_]^+^ intermediate. Pr: propanyl, bpy: 2,2′-bipyridine.

Electrochemiluminescence (ECL) is a fundamental process in electrochemistry and plays an important role in the design of biosensors for applications in clinical diagnosis.^32,33^ Tris(2,2′-bipyridine) ruthenium(ii) and its derivatives are a group of widely used ECL reagents.^34^ However, the elucidation of the mechanism of ECL is challenging. Besides the traditional mechanism (Fig. S4†),^32^ Bard *et al.* ^35^ have proposed a new mechanism for the tris(2,2′-bipyridine) ruthenium(ii)/tri-*n*-propylamine (TPrA) system (Fig. 3a) when smaller potential is applied, based on the results of scanning electrochemical microscopy (SECM)-ECL experiments. Here, we try to apply the setup proposed in this work to detect key intermediates under similar conditions to confirm the proposed mechanism. The mixed solution of 1 ppm tris(2,2′-bipyridine) ruthenium(ii) and 10 ppm tripropylamine (10 mM NH_4_Cl as the supporting electrolyte) was used in the experiment. As shown in Fig. 3b–d, when 0.8 V was applied, the intermediate ions of [Pr_2_N = CHCH_2_CH_3_]^+^, [NHPr_2_]^+^ and Ru(bpy)_3_^+^ could be successfully detected and Ru(bpy)_3_^3+^ was absent, whereas if no potential was applied, all of these intermediate ions were absent in the mass spectra. When a higher potential of 1.3 V was applied, Ru(bpy)_3_^3+^ could be detected (Fig. S5†) with the absence of Ru(bpy)_3_^+^. These results evidence that the mechanism of the ECL has two routes. Under lower potential (0.8 V) conditions, only TPrA is oxidized on the electrode and no Ru(bpy)_3_^3+^ is produced, and a Ru(i) complex is a key intermediate (Fig. 3a). Under a higher potential (1.3 V), Ru(iii) is a key intermediate, which will be reduced to Ru(ii)* by a co-reactant to generate luminescence (Fig. S4†). These conclusions are consistent with what Bard *et al.* have proposed. To our best knowledge, this is the first on-line MS investigation of ECL mechanisms. Clearly, we have shown that complicated reaction pathways in organometallic EC reactions can also be studied by the EC/MS setup proposed in this work.

The detection of intermediates of very short life-time is important for offering profound insight into reaction mechanisms. Owens *et al.* ^36^ measured that in the electrochemical oxidation of uric acid, the diimine intermediate (Fig. S6a†) has a half-life of about 23 ms. Brown *et al.* ^19^ detected successfully this diimine intermediate by their water-wheel shaped EC/MS setup. To test whether this fleeting intermediate can be detected by our setup, 100 ppm uric acid solution (10 mM NH_4_Cl as the electrolyte) was used, and the experiment was conducted as aforementioned. When no potential was applied, the uric acid molecular ion *m*/*z* 167 was detected in the negative ion mode (Fig. S6b†). When a potential of 1.0 V was applied, the diimine intermediate *m*/*z* 165 was detected (Fig. S6c†). The results obtained here are consistent with previous reports.^19^ Different from the dopamine example, here the uric acid negative ion can also be detected. Though different potentials (0.5 to 3.0 V) were applied, the complete conversion of uric acid was not achieved (Fig. S7†), which may be attributed to the strong adsorption of UA on the carbon electrode. Similar to the dopamine example, by switching the applied potential on and off on each occasion, a single pulse by the piezoelectric pistol was triggered; the spectra corresponding to uric acid only and the diimine intermediate with the uric acid also changed accordingly (Fig. S6d and e†). This example verifies the sampling model depicted in Fig. S3,† and also demonstrates that reaction intermediates of very short life-time can be detected by this method.

Compared to similar EC/MS methods, our design has the following merits. (1) Our device is small, easy to fabricate, and can readily be coupled to most commercial mass analyzers, which is convenient for widespread applications. (2) Sampling directly from the electrode surface and the ultra-short distance (about 2 mm) of the electrode surface to the MS inlet can make detection and characterization of fleeting intermediates possible, thus facilitating establishment or confirmation of reaction mechanisms. (3) Due to the tip of the hybrid ultramicroelectrode directly pointing towards the MS inlet and the ultra-short distance between them, high sampling efficiency has been achieved. (4) Girod *et al.* ^37^ have reported that the droplet size has a significant influence on the reaction kinetics. The feature of single droplet sampling in our method can make studies of size-dependent reaction acceleration in tiny droplets possible.^38–41^

## Conclusions

We report a novel EC/MS setup for on-line monitoring of electrochemical reactions on the ultramicroelectrode surface. A hybrid ultramicroelectrode is directly used as a micro-electrochemical cell and a nano-spray emitter. The ionization process is initiated by a hand triggered piezoelectric pistol. Dopamine and uric acid oxidations, as well as ruthenium(ii) ECL reactions are studied as proof-of-principle examples. Key intermediates have been detected to confirm proposed mechanisms, and a short-lived intermediate has also been identified. This work provides a convenient way to study complicated electrochemical reactions, facilitating elucidation of reaction mechanisms.
