# Supplementary material for: Mass spectrometric snapshots for electrochemical reactions
Source: Chem Sci. 2016 Jul 6;7(11):6684–8. doi: 10.1039/c6sc01978a (PMC5355862; doi:10.1039/c6sc01978a)
Supplement: Supplementary file 1 [file SC-007-C6SC01978A-s001.pdf]

Supporting Information for

Mass Spectrometric Snapshots for Electrochemical Reactions

Ran Qiu[+], Xin Zhang[+], Hai Luo[*], and Yuanhua Shao[*]

Beijing National Laboratory for Molecular Science
College of Chemistry and Molecular Engineering
Peking University
202 Chengfu Road, Beijing 100871, China
E-mail: hluo@pku.edu.cn and yhshao@pku.edu.cn

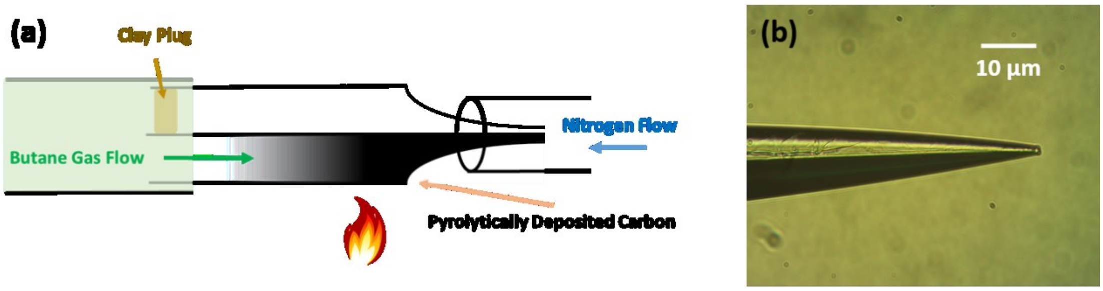

Fig. S1. (a) Schematic illustration of fabrication method of the hybrid ultramicroelectrode. Pressure of butane is 0.15 MPa, pressure of nitrogen gas is 0.02 MPa, heat time is 30s.[S1] (b) Photograph of the hybrid ultramicroelectrode under microscopy.

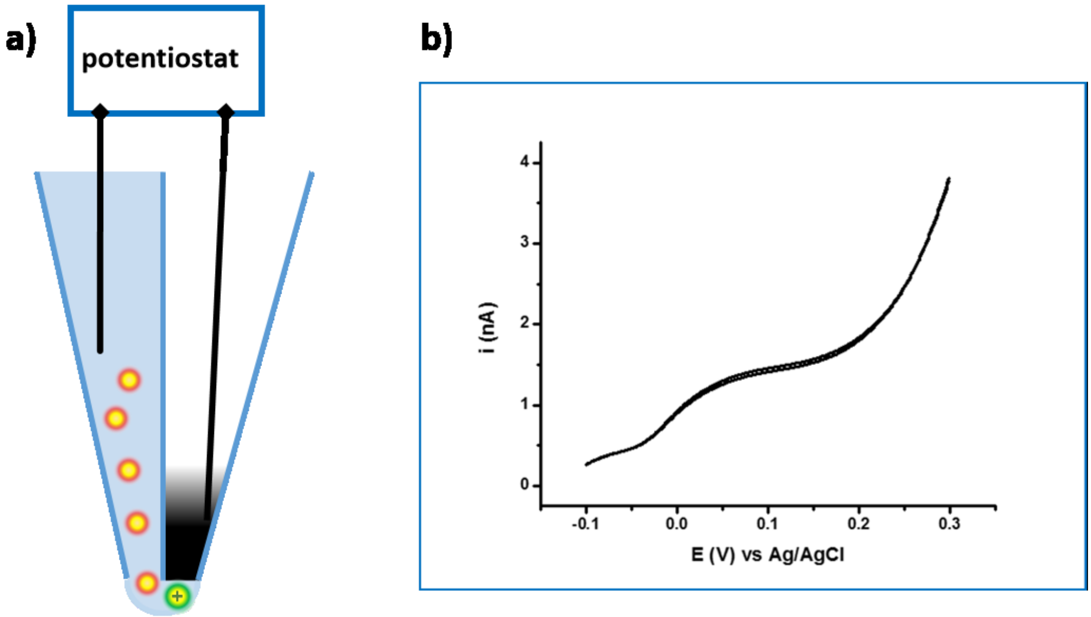

Fig. S2. (a) Schematic illustration of micro-electrochemical cell in the hybrid ultramicroelectrode. (b) Cyclic voltammogram of 2 mM dopamine in the hybrid ultramicroelectrode, 10 mM KCl, scan rate: 20 mV/s. The pipettes were filled with aqueous solution from the back using a small (10 µL) syringe. The cyclic voltammogram was recorded using a CHI 910B electrochemical workstation with a Faraday cage.

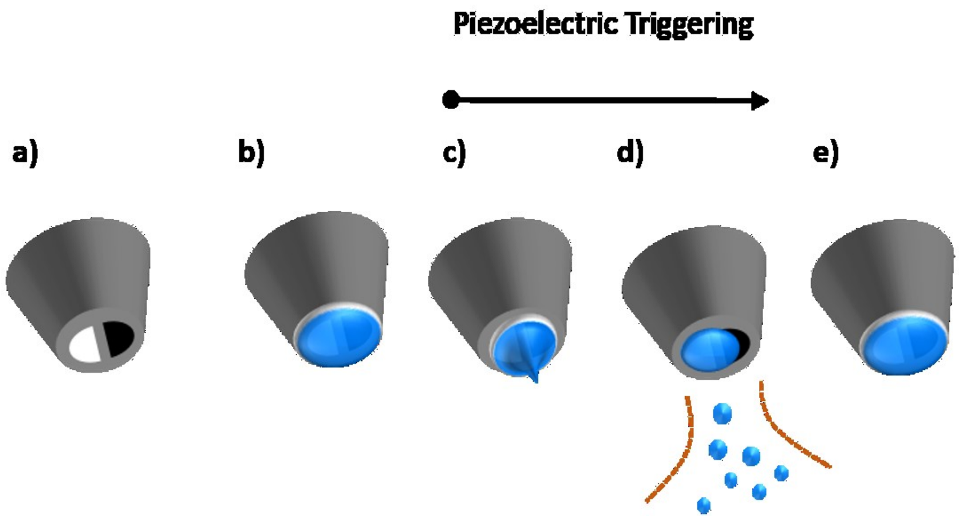

Fig. S3. (a) Illustration of the hybrid electrode tip. One barrel is default, and the other is filled with carbon by pyrolytic deposition. (b) Thin liquid layer formation on the tip. (c) Taylor cone formation when piezoelectric triggering starts. (d) Single-droplet explosion into smaller droplets for desolvation and subsequent ionization. (e) Another single-droplet is formed. The cycle starts again from c to e.

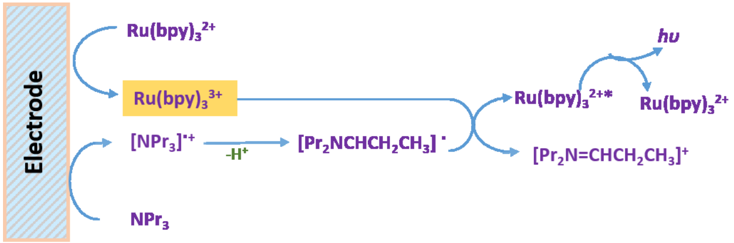

Fig. S4. Traditional ECL mechanism of $Ru(bpy)_3^{2+}$/TPrA system.[S2] The key intermediate in the traditional mechanism is the $Ru(bpy)_3^{3+}$, which is different from that in the Bard's mechanism (shown in Fig 3a in the main text).

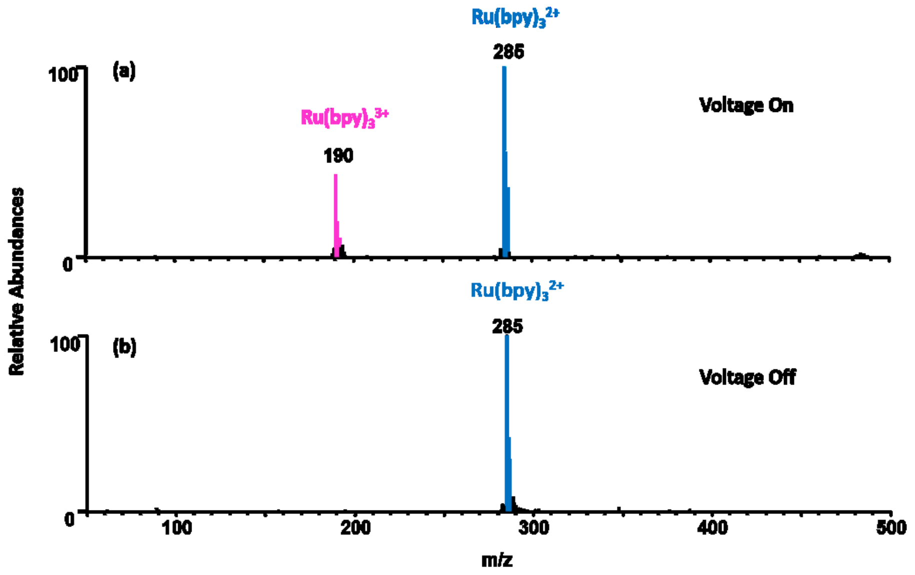

Fig. S5. Mass spectra of the Ru-TPrA system. (a) Potential of 1.0 V was applied to the EC system, the Ru(III) complex (m/z 190) as the ECL intermediate can be detected. (b) When no potential was applied, only the Ru(II) complex (reactant) was detected.

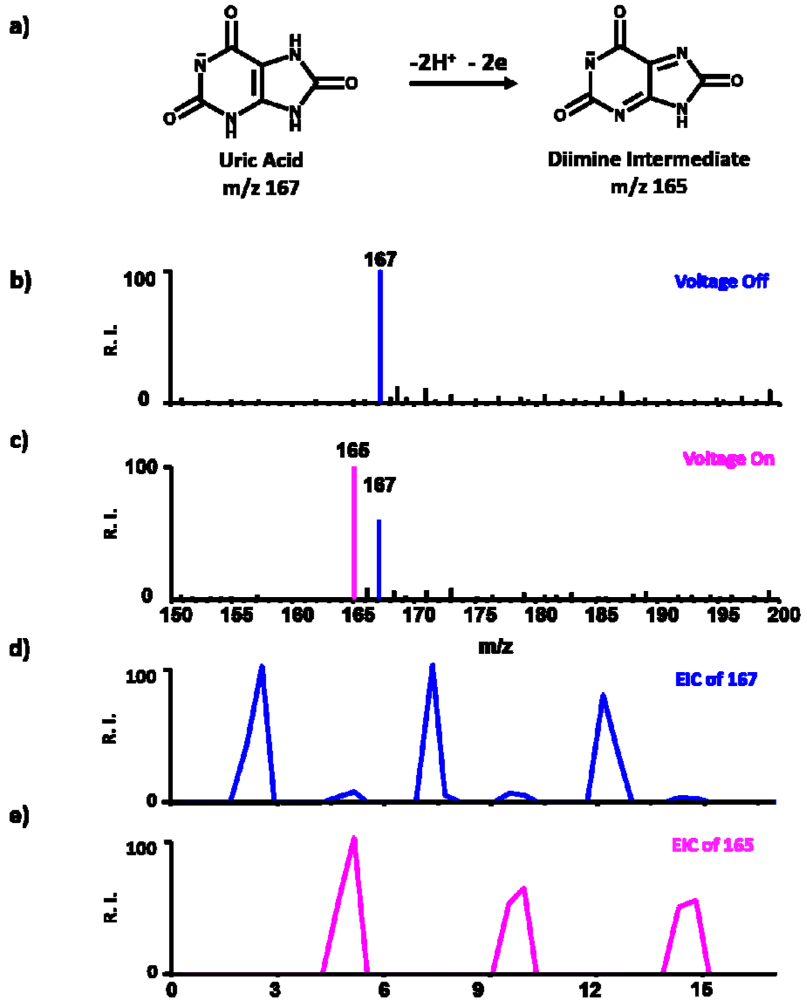

Fig. S6. Mass spectra of 100 ppm uric acid (10 mM NH_4Cl as the supporting electrolyte). (a) Uric acid oxidation reaction. The diimine intermediate has a half-life of about 23 ms. (b) Mass spectrum when applied potential was 0 V. (c) Mass spectrum when potential of 1.0 V was applied. (d-e) 6 triggers were continuously shot. The 1st, 3rd, and 5th triggers were shot when the potential was off. The 2nd, 4th, and 6th triggers were shot when the potential was on. (d) The extracted ion chromatogram of m/z 167 during the 6 shots. (e) The EIC of m/z 165 during the 6 shots.

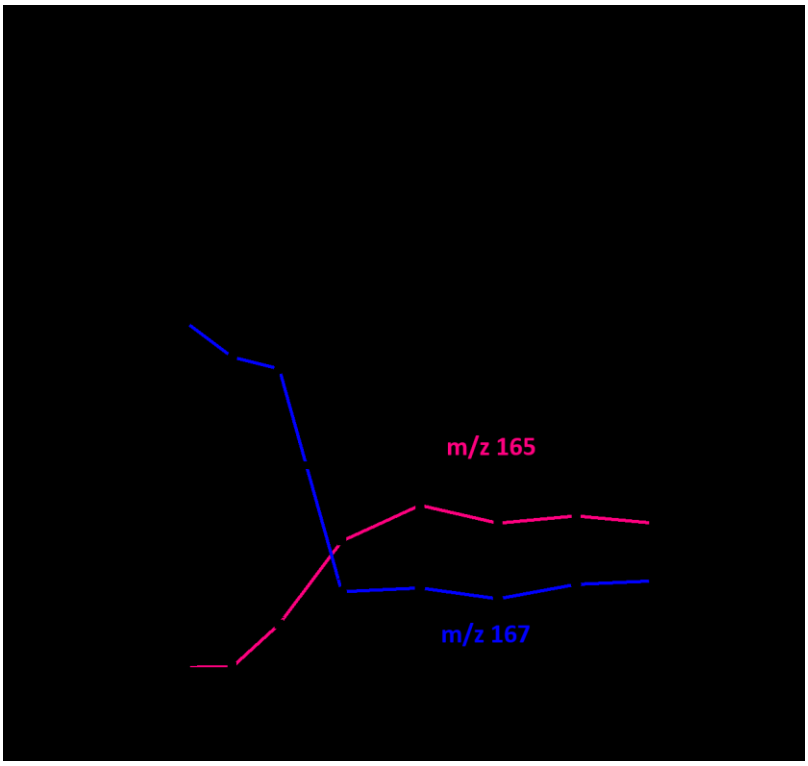

Fig. S7. The changes of signal intensity of m/z 167 (uric acid) and m/z 165 (diimine intermediate) in MS when different potentials are applied. Note that the uric acid is not completely consumed in the potential range applied. 100 ppm uric acid, 10 mM NH_4Cl.

Reference

S1 Y. Takahashi, A. I. Shevchuk, P. Novak, Y. J. Zhang, N. Ebejer, J. V. Macpherson, P. R. Unwin, A. J. Pollard, D. Roy, C. A. Clifford, H. Shiku, T. Matsue, D. Klenerman, Y. E. Korchev, *Angew. Chem. Int. Ed.* 2011, **50**, 9638-9642.

S2 I. Rubinstein, A. J. Bard, *J. Am. Chem. Soc.* 1981, **103**, 512-516.
